# Hollow nitrogen-containing core/shell fibrous carbon nanomaterials as support to platinum nanocatalysts and their TEM tomography study

**DOI:** 10.1186/1556-276X-7-165

**Published:** 2012-03-02

**Authors:** Cuifeng Zhou, Zongwen Liu, Xusheng Du, David Richard Graham Mitchell, Yiu-Wing Mai, Yushan Yan, Simon Ringer

**Affiliations:** 1 Australian Centre for Microscopy and Microanalysis, The University of Sydney, New South Wales, 2006, Australia; 2 Centre for Advanced Materials Technology, School of Aerospace Mechanical and Mechatronic Engineering J07, The University of Sydney, New South Wales, 2006, Australia; 3 Chemical and Biomolecular Engineering, University of Delaware, Newark, DE, 19716, USA

**Keywords:** carbon nanofiber, N-doping, core/shell, polyaniline, catalyst support, methanol oxidation, TEM tomography

## Abstract

Core/shell nanostructured carbon materials with carbon nanofiber (CNF) as the core and a nitrogen (N)-doped graphitic layer as the shell were synthesized by pyrolysis of CNF/polyaniline (CNF/PANI) composites prepared by *in situ *polymerization of aniline on CNFs. High-resolution transmission electron microscopy (TEM), X-ray diffraction (XRD), Fourier transform infrared and Raman analyses indicated that the PANI shell was carbonized at 900°C. Platinum (Pt) nanoparticles were reduced by formic acid with catalyst supports. Compared to the untreated CNF/PANI composites, the carbonized composites were proven to be better supporting materials for the Pt nanocatalysts and showed superior performance as catalyst supports for methanol electrochemical oxidation. The current density of methanol oxidation on the catalyst with the core/shell nanostructured carbon materials is approximately seven times of that on the catalyst with CNF/PANI support. TEM tomography revealed that some Pt nanoparticles were embedded in the PANI shells of the CNF/PANI composites, which might decrease the electrocatalyst activity. TEM-energy dispersive spectroscopy mapping confirmed that the Pt nanoparticles in the inner tube of N-doped hollow CNFs could be accessed by the Nafion ionomer electrolyte, contributing to the catalytic oxidation of methanol.

## Introduction

The supporting materials of electrochemical catalysts have been shown to have great effects on the electrochemical performance of the catalysts [1]. Many kinds of carbon nanomaterials, such as carbon black, carbon nanotubes (CNTs) and graphenes, have been studied as supports for platinum (Pt)-based electrochemical catalysts [2-5]. Conductive polymers are another type of catalyst supports and are believed to provide both electron and proton conductions in the catalyst layer of the electrode of a fuel cell [6-9]. It was concluded that polyaniline (PANI) in the catalyst in the anode was beneficial in the absorption of water and formation of an active oxy-compound Pt-OH_ads_, which would promote oxidizing CO to CO_2 _and Langmuir absorption of methanol [10]. Recently, nitrogen (N)-doped carbon materials seemed promising catalyst supports through the effects of N-doping on surface physicochemical properties, electron transfer and nanostructures of the supports and catalysts, which exhibited higher catalytic activity and durability [11-15]. Two methods have been usually employed for the synthesis of N-doped carbon nanomaterials: by direct N-doping during preparation of the carbon nanomaterials and treatment of the carbon composites with N-containing precursors [11-19]. PANI could be a good candidate for such precursors owing to the facile post-treatment for its carbonization [14,15,18-20]. Also, it exhibits a strong interaction with aromatic graphenes in carbon nanomaterials, which facilitates easy fabrication of uniform nanostructured carbon/PANI composites. Moreover, Pt catalysts supported by CNT/PANI composites have been demonstrated to have excellent electrochemical activity for methanol oxidation [21-24]. Thus, it is expected that the electrochemical catalytic performance of catalysts supported by carbon/PANI composites can be further improved by N-doping of the composite supports followed by carbonization. In this work, a simple method to prepare N-doped carbon nanofibers (CNFs) by carbonization of preformed PANI was developed. It was found that the carbonization treatment of the CNF/PANI composites greatly increased the electrochemical catalytic activity for methanol oxidation in fuel cells. Transmission electron microscope (TEM) tomography was used to examine the dispersion of Pt nanoparticles in the catalyst supports.

### Experimental details

#### *In situ *polymerization to prepare CNF/PANI core/shell composites

CNF (Pyrograf Products Inc., Cerdaville, OH, USA) was first treated with a conventional chemical oxidation method using concentrated nitric acid to remove the metal impurities. The CNF/PANI nanocomposite was prepared using the following procedure. A given amount of carbon nanofiber powder obtained above and 1 ml aniline were put into 50 ml 1 mol/L HCl solution (weight ratio between CNF and aniline was 2:3). Then, 2.5 g FeCl_3_.6H_2_O in 20 ml solution was added into the mixture. The mixture was constantly stirred for 24 h. After filtration, the precipitate was washed with 1 M HCl, water and, finally, ethanol. The product was dried at 80°C for 12 h and stored in a desiccator before characterization. The CNF/PANI composite was treated at 900°C in an oven for 5 min to carbonize the PANI. The heat-treated and untreated CNF/PANI supports in this study are denoted as CNF/HPANI and CNF/PANI, respectively.

#### Nanocatalyst fabrication and the methanol oxidation

Pt particles for dispersion on the catalyst supports were prepared by a method of *in situ *reduction of a Pt salt with formic acid. H_2_PtCl_6_.H_2_O (0.2 g) was dissolved in 10 ml distilled water. CNF/HPANI catalyst support was dispersed in the distilled water by sonication for 20 min. Then, an appropriate amount of H_2_PtCl_6_.H_2_O solution was added dropwise to the above support suspension with sonication. After adding formic acid, the suspension was heated in a water bath at 70°C for 3 h with ultrasonication to complete the reduction of Pt. After centrifugation and washing with water and ethanol, the material was dispersed in 0.05 wt.% Nafion solution and sonicated for 20 min to prepare the catalyst ink. An amount (3.5 μL) of this catalyst solution was added to the surface of the glassy carbon (GC) electrode (3 mm in diameter) and dried in air. For comparison, the catalyst with CNF/PANI composite as the support was also prepared under the same experimental conditions. Energy dispersive spectroscopy (EDS) analysis indicated that the Pt content in both catalysts was approximately 30 wt.%. Prior to the electrochemical measurement, the catalyst-covered electrode was soaked in the electrolyte solution (0.5 M H_2_SO_4_) for 10 min. The electrochemical activity of the catalyst with respect to methanol oxidation was tested in 1 M CH_3_OH + 0.5 M H_2_SO_4_.

#### Characterization and measurements

X-ray diffraction (XRD) patterns were obtained using an X-ray diffractometer (Siemens D5000, Siemens, AG, Munich, Germany) with Ni-filtered CuKα radiation (*λ *= 1.54 Å). Scanning electron microscopy (SEM) (Zeiss ULTRA plus, Carl Zeiss AG, Oberkochen, Germany)) was used to observe the morphology of the samples. Transmission electron microscopy (TEM) was done with the following instruments: routine imaging, Philips CM12 (120 kV) (Philips, Eindhoven, The Netherlands); high-resolution TEM (HRTEM) and EDS microanalyses, JEOL 2200FS (200 kV) (JEOL Pty. Ltd., Frenchs Forest, New South Wales, Australia ) and electron tomography, JEOL 1400 (120kV) (JEOL Pty. Ltd.). Fourier transform infrared (FTIR) attenuated total reflection spectra and UV-visible spectroscopy (UV-Vis) spectra were recorded on a Bruker IFS66V FT-IR (Bruker Optics, Melbourne, Victoria, Australia) and a Cary 5-UV-Vis spectrometer (Agilent Technologies, Santa Clara, CA, USA), respectively. Raman spectroscopy of the samples was recorded with an inVia Renishaw Raman (Renishaw Oceania Pty. Ltd., Melbourne, Victoria, Australia) using a He-Ne laser at 633 nm wavelength. The electrochemical characterizations were performed on a CHI1202A Electrochemical Analyzer (CH Instruments Inc., Austin, TX, USA). All the solutions were prepared in distilled water, and all potentials reported were referenced to a saturated calomel electrode (SCE). A three-electrode electrochemical cell was used for the measurements, where the counter electrode was a Pt foil and the reference electrode was a SCE.

The 2D experimental images for the 3D TEM (electron tomography) were recorded in a bright-field mode on a JEOL 1400 (120 kV) using a high-tilt sample holder. The tilt series were acquired with automatic rectification (corrections of focus and horizontal displacement) by using the SerialEM [25] softwareon a 1,350 × 1,040 pixel Erlangshen CCD camera (Gatan, Inc., Pleasanton, CA, USA). The tilt range was set from -65° to 65° with a basic increment of 1°, giving a total of 130 images. The tilt series data were treated for image processing and reconstruction using the IMOD software program from Boulder Laboratory for 3D Electron Microscopy of Cells and the University of Colorado [26].

## Results and discussion

**Figure 1:**
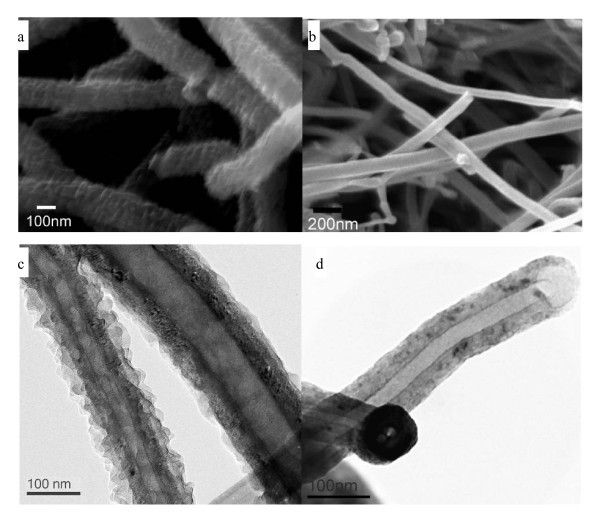
**Scanning electron microscopy (SEM) and transmission electron microscopy (TEM) images**. SEM micrographs of (**a**) carbon nanofiber/polyaniline (CNF/PANI) and (**b**) CNF; TEM bright-field micrographs of (**c**) CNF/PANI and (**d**) CNF/HPANI.

A typical SEM image of the CNF/PANI samples is shown in Figure 1a, where the CNF/PANI composites exhibit a fibrous morphology with diameters in the range of 100 to 200 nm, which are obviously larger than those of the CNFs (50 to 150 nm as given in Figure 1b). The term 'CNF' was used by the manufacturer to avoid confusion with the conventional carbon nanotubes whose diameter was much less than 100 nm. The surface of the fibrous composite is also rougher than the CNF, confirming the coating of PANI. The TEM image (Figure 1c) reveals a coaxial structure in which the CNF is wrapped by a PANI layer with a thickness less than 100 nm. Note that the CNFs have a hollow core and a larger diameter than conventional MWCNTs. After the carbonization treatment, the CNF/PANI fibrous composite becomes thinner (Figure 1d), and a porous shell seems to form from the decomposition of the PANI shell, as shown in the high-magnification TEM image (Figure S1b [see Additional file 1]). EDS line scan analysis (the direction was indicated by arrows in Figure S1c, d, respectively) along the cross-section of the CNF/HPANI (Figure S1d) indicates a lesser N content than that in CNF/PANI (Figure S1c). The shoulder peaks on both sides of the carbon line scan curve clearly indicate the existence of the PANI layers (Figure S1c). Due to the absence of the CNF core in the edge of the CNF/PANI core/shell structure, the content of N in these areas is a little higher than that in the central area. The shoulder peak on the right side of the carbon line scan curve of the CNF/HPANI shows the N-containing carbon layer from the carbonization of the PANI shell. As only 0.24 wt.% of N existed in the HPANI/CNF (Figure S2 and Table S1), the intensity of N in Figure S1c, d was multiplied by ten to make the line clear.

**Figure 2:**
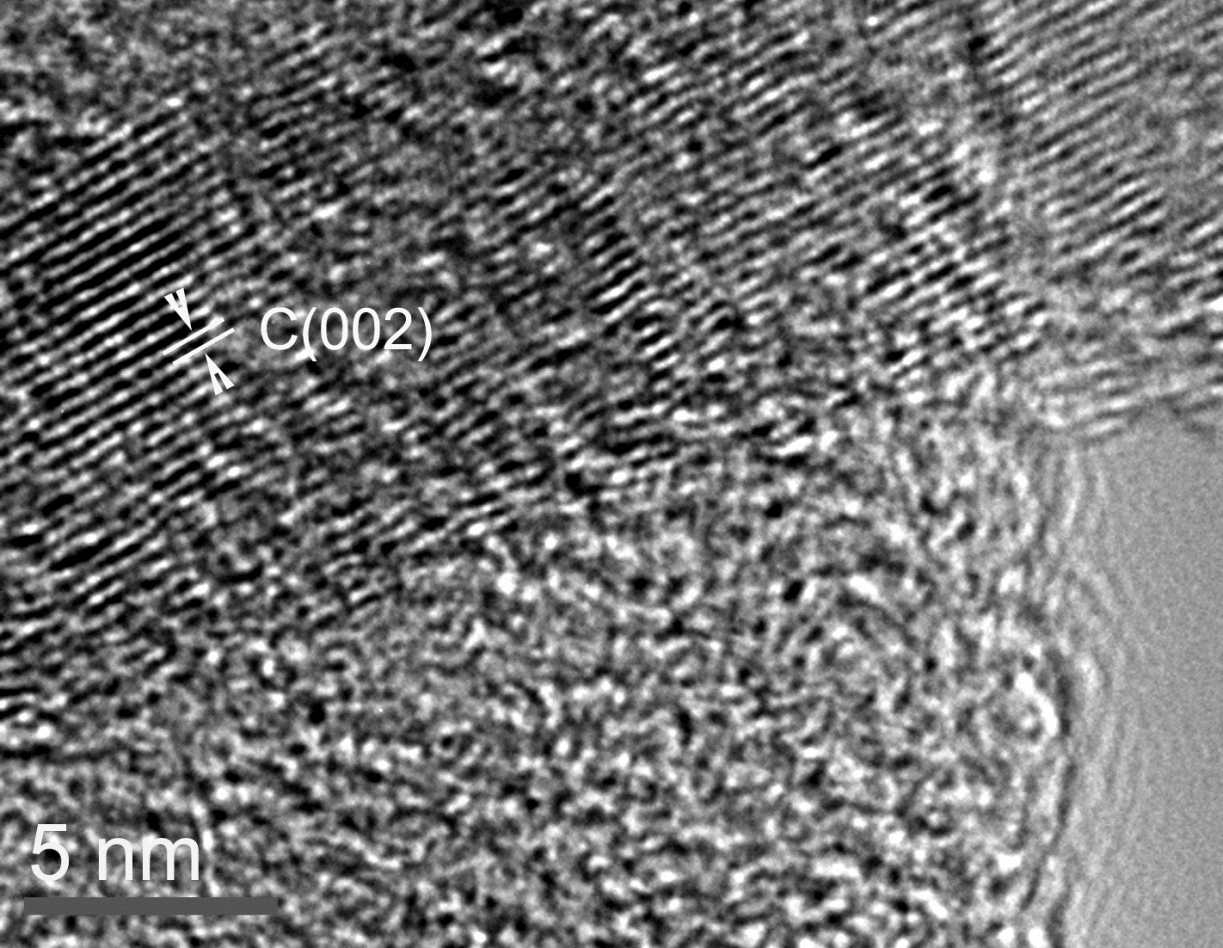
**HRTEM image of CNF/HPANI**.

In the HRTEM image of the carbonized sample (Figure 2), there is a clear boundary between the CNF core and the carbonized shell due to the difference in electron transmission in these two layers of the carbon materials. Comparing the inner tube-wall of the CNF core (the upper darker area) to the carbonized PANI shell (the lower light area), the N-doped carbon surface appears to have an amorphous structure and contains a disordered graphitic phase [27]. This may provide active sites for easy anchoring of Pt nanoparticles [15]. This is because nitrogen is likely to serve an additional or separate role by improving the ability of graphenes to donate electrons [28], which can then interact with the Pt particles.

**Figure 3:**
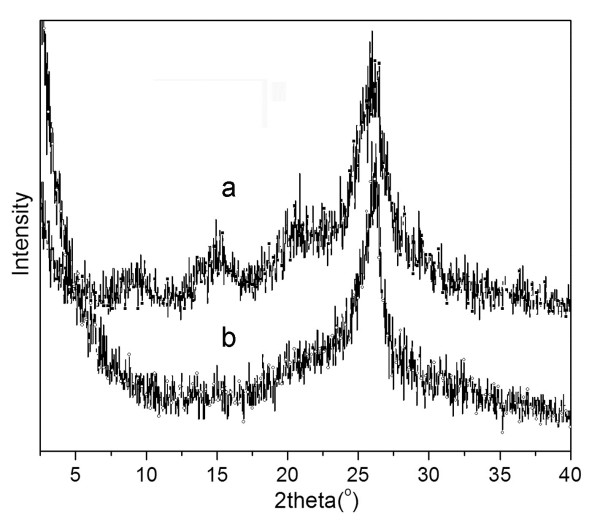
**X-ray diffraction patterns of (a) CNF/PANI and (b) CNF/HPANI**.

The carbonization of the PANI layer in the composite was also confirmed by XRD, FTIR and Raman studies. Figure 3 shows the X-ray diffraction patterns from the CNF/PANI and CNF/HPANI composites, respectively. In the XRD patterns of the CNF/PANI (Figure 3, curve a), four broad peaks around 2*θ *= 9° (001), 14.6° (011), 20.3° (100) and 25.8° (110) are observed, which are due to the PANI layers. The peak centered at 2*θ *= 20.3° can be attributed to the periodicity parallel to the polymer chain, while that at 2*θ *= 25.8° may originate from the periodicity perpendicular to the polymer chain. The peak at 25.8° is stronger than that at 20.3°, which is similar to that of the highly doped emeradine salt (ES) [29,30]. After carbonization, the XRD pattern of the composite (Figure 3, curve b) is completely changed, and the peaks from the PANI all disappeared. The only peak seen at 26.3° is typically from (002) of the carbon materials, confirming the complete carbonization of the PANI shell in the composites.

**Figure 4:**
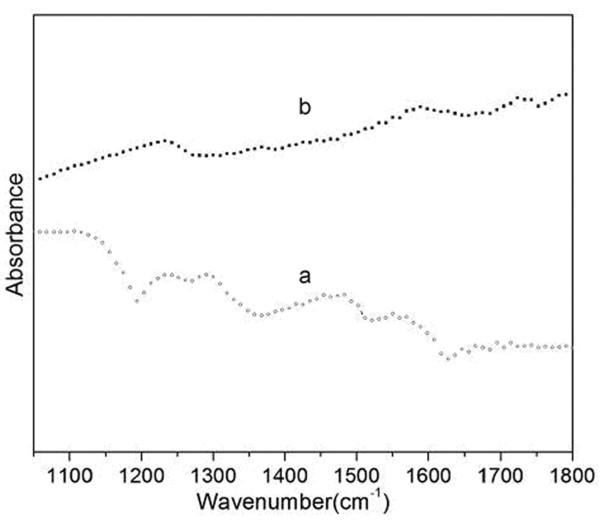
**FTIR spectra of (a) CNF/PANI and (b) CNF/HPANI**.

The FTIR spectrum of the CNF/PANI composite (curve a in Figure 4) shows typical PANI absorption bands. The four strong bands at 1,560, 1,483, 1,293 and 1,243 cm^-1 ^can be attributed to the C = C stretching vibration mode of the quinonoid and benzenoid rings, and the stretching mode of C-N and the protonated C-N group, respectively [31,32], suggesting that the PANI shell in the composite is in the ES form. The FTIR spectrum of the CNF/HPANI composite (curve b in Figure 4) is quite different from that of the CNF/PANI, and typical bands from the PANI have disappeared due to the carbonization of the PANI macromolecule [20]. The new peaks at 1,235, 1,587 and 1,720 cm^-1 ^originate from OH, C = C and C = O, respectively, indicating the presence of carbonyl and/or carboxyl groups in the carbonized products. These may increase the hydrophilicity of the CNF/HPANI and facilitate the absorption of Pt nanocatalysts in the ensuing impregnation process, thus enhancing the distribution and long-term performance of the resulted catalysts.

**Figure 5:**
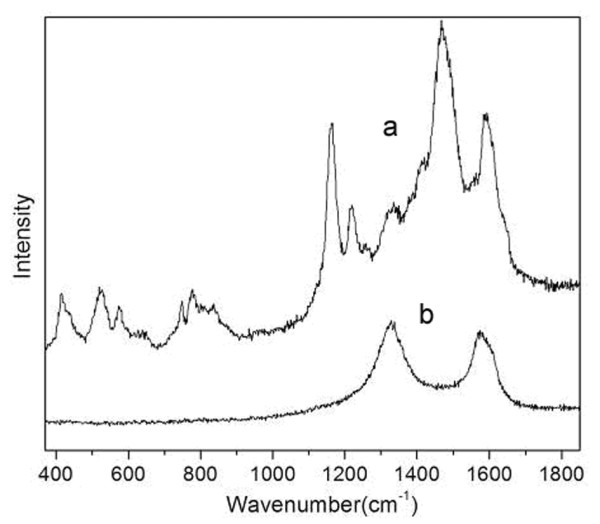
**Raman spectra of (a) CNF/PANI and (b) CNF/HPANI**.

Raman spectroscopy is very sensitive to the changes in the structure of these materials arising from carbonization. For the Raman spectrum of the CNF/PANI composite (curve a in Figure 5), several strong peaks from the C-H bending of the quinoid ring at 1,160 cm^-1^, C = C stretching of the quinoid ring at 1,460 cm^-1 ^and stretching of the benzenoid ring at 1,590 cm^-1 ^are observed, hence confirming that the PANI shell has a doped ES structure [32]. After carbonization, the Raman spectrum transformed to a simple pattern (curve b in Figure 5) showing two strong peaks at 1,335 and 1,595 cm^-1 ^(Figure 5). These two peaks are referred to as the D and G bands for carbonaceous materials, respectively [32,33]. The D band is slightly stronger than the G band, indicating the existence of a larger amount of disordered graphitic phase in the CNF/HPANI.

**Figure 6:**
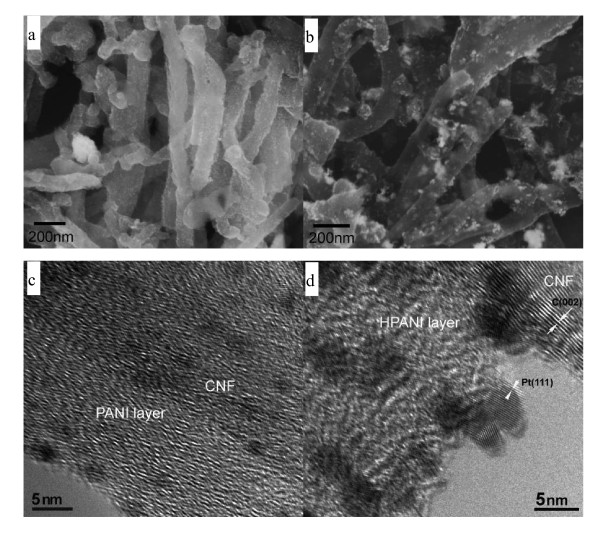
**Scanning electron microscopy (SEM) and high-resolution transmission electron microscopy (HRTEM) micrographs**. SEM micrographs of (**a**) carbon nanofiber/polyaniline-supported platinum (CNF/PANI-Pt) and (**b**) carbon nanofiber/carbonized polyaniline-supported platinum (CNF/HPANI-Pt); HRTEM micrographs of (**c**) CNF/PANI-Pt and (**d**) CNF/HPANI-Pt.

Figure 6 shows the morphologies of the CNF/PANI-Pt and CNF/HPANI-Pt catalyst materials. The light-colored grains on the surface of the tube-like particles in the SEM image (Figure 6a) are fine Pt nanoparticles. They were uniformly dispersed on the surface of the CNF/PANI. The core/shell structure of the CNF/PANI with the PANI nanolayer appeared to promote a uniform dispersion of Pt particles over the catalyst supports, as shown in the HRTEM image (Figure 6c). In contrast, with the CNF/PANI-Pt sample, significant clustering of the Pt particles is present on the surface of the CNF/HPANI in the SEM image (Figure 6b). HRTEM imaging confirmed that the clusters seen in the SEM are indeed Pt since Pt (111) lattice fringes can be seen (Figure 6d). The size of Pt nanocrystals is in the range 2 to 6 nm.

**Figure 7:**
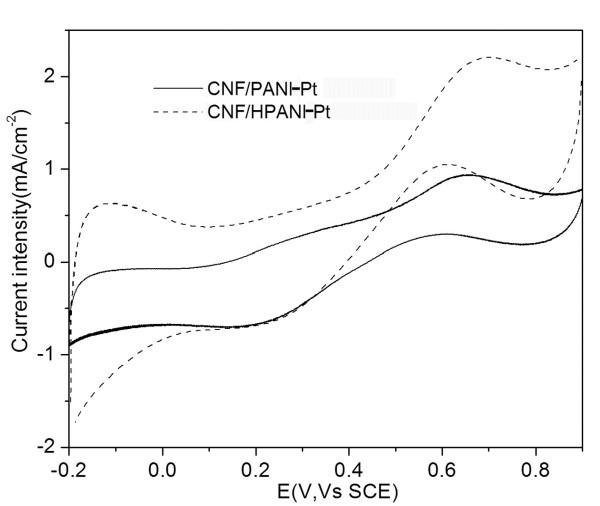
**Cyclic voltammograms (tenth cycle) of CNF/PANI-Pt and CNF/HPANI-Pt**. In 1 M CH_3_OH + 0.5 M H_2_SO_4_. Scan rate, 50 mV/s.

**Figure 8:**
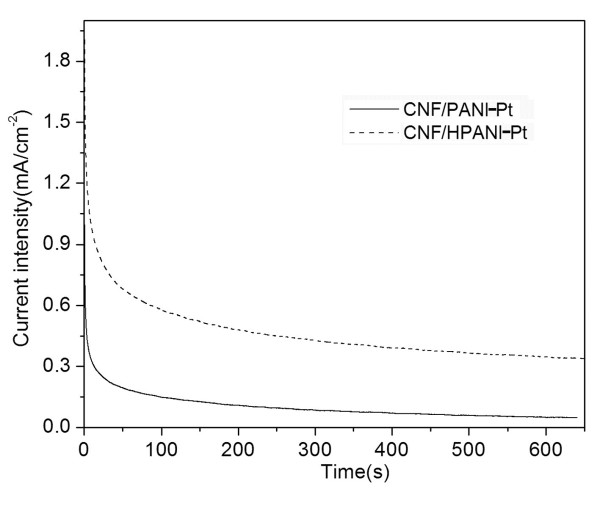
**Chronoamperograms**. CNF/PANI-Pt and CNF/HPANI-Pt at 0.6V in 1 M CH_3_OH + 0.5 M H_2_SO_4_. a and b were deleted from the caption.

Figure 7 illustrates the electrochemical catalytic activity of the CNF/PANI-Pt catalyst towards oxidation of methanol in which the forward current peak is attributed to the oxidation of CH_3_OH molecule, and the backward current peak to the oxidation of the adsorbed intermediates [21-23,34]. Obviously, the methanol oxidation current intensity on the CNF/HPANI-Pt catalyst is much larger than that on the CNF/PANI-Pt catalyst, indicating the beneficial effects of carbonization of PANI to fabricate catalyst supports in fuel cells. Figure 8 shows the chronoamperograms for the oxidation of methanol on the CNF/PANI-Pt and CNF/HPANI-Pt catalyst-covered GC electrode in acidic media at a potential of 0.6 V. The current density shows sharp decay in activity on both catalyst-covered electrodes in the first 100 s, which could be related to the adsorbed intermediate products of methanol oxidation on the surface of the fresh Pt catalysts. After that, the current density generally reached a steady state. The current density of methanol oxidation on the CNF/HPANI-Pt catalyst is much higher than that covered with the CNF/PANI-Pt catalyst throughout the whole chronoamperogram test. In the nearly steady state, for instance, the current density of methanol oxidation at 600 s on the CNF/HPANI-Pt catalyst is approximately seven times of that on the CNF/PANI-Pt. The higher current intensity for the methanol oxidation on the catalyst with the CNF/HPANI composite as support is believed to be related to its core/shell carbon fibrous nanostructure with N-doping.

The location of the metal catalyst nanoparticles in the catalyst support has a great influence on their catalytic performance. From the SEM images, comparing with the CNF/PANI (Figure 6a), a much greater degree of Pt nanoparticle agglomeration is observed on the CNF/HPANI supports (Figure 6a, b); however, the catalytic performance of this carbonized material is much better than that of the CNF/PANI as shown in Figures 7 and 8. So, it is expected that the location of the Pt nanoparticles in the two supports may be different.

**Figure 9:**
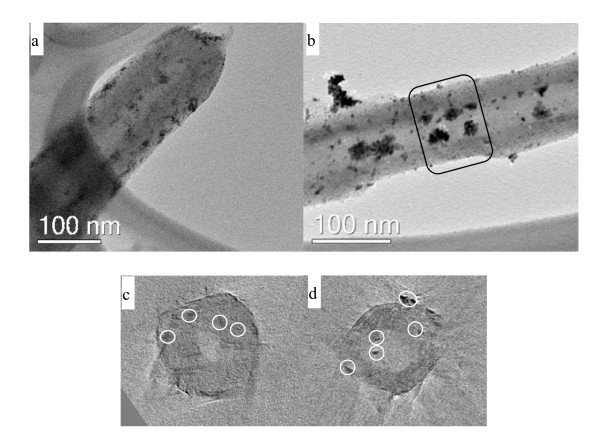
**2D transmission electron microscopy (TEM) bright-field images**. One of the 2D TEM bright-field images from the tilt series used for 3D TEM analysis of the samples: (**a**) carbon nanofiber/polyaniline-supported platinum (CNF/PANI-Pt) and (**b**) carbon nanofiber/carbonized polyaniline-supported platinum (CNF/HPANI-Pt) used to reconstruct their volume, and the transverse sections through the volume obtained by reconstruction of the (**c**) CNF/PANI-Pt and (**d**) CNF/HPANI-Pt.

TEM electron tomography was used to study the dispersion of the Pt nanoparticles on the catalyst supports since it is a suitable technique that can clearly determine the exact location of the particles with respect to tube-like nanomaterials. Conventional TEM images are a 2D projection of a 3D structure, which, therefore, cannot provide 3D information without tilting the specimen [35]. Typical 2D TEM images of these two catalysts are shown in Figure 9a, b. Also shown are the respective transverse sections obtained from the reconstructed volumes derived from electron tomograms (Figure 9c, d). In Figure 9c, some small aggregation of the Pt nanoparticles (enclosed in circles) were observed in the PANI shell in the CNF/PANI support plus many particles on the outer surface of the PANI shell. It is believed that the embedded Pt nanocatalysts in the PANI layers may be unable to contribute to the electrochemical catalytic performance owing to the obstruction of the surrounding PANI. While in the transverse section of the CNF/HPANI-Pt (Figure 9d), several aggregations of Pt nanoparticles (enclosed in circles) were found either on the inside of the inner tube-wall or on the outer surface of the tube.

**Figure 10:**
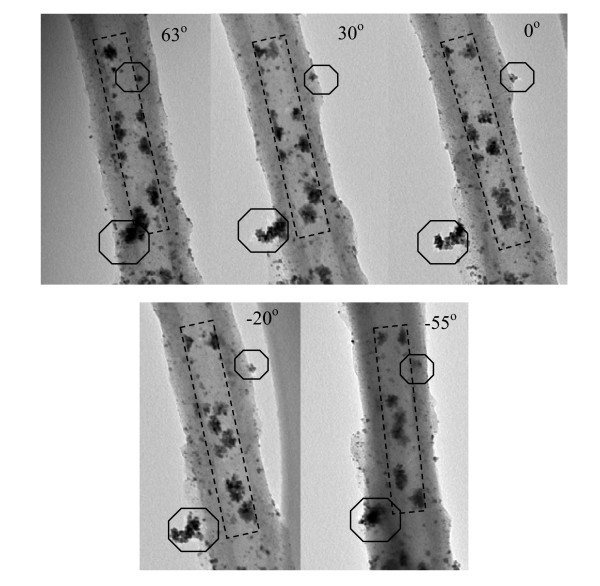
**Selected bright-field images of the TEM tilt series**. Obtained by tilting the CNF/HPANI-Pt sample (same area as Figure 9b) to different angles: +63°, +30°, 0°, -20° and -55°.

TEM images taken during rotation of one tube about its axis provided detailed information on the locations and shape of the Pt nanoparticles inside/outside the tube by following the movement. Selected images of TEM tilt series from the CNF/HPANI-Pt show the same area but with different perspectives due to tilting the sample from +65° to -65° (Figure 10). Clearly, the particles inside the tube (enclosed in a rectangle) moved steadily around the tube axis as the tube was tilted continuously, but their motion was restricted within the inner tube; while the particles (enclosed in octagons) that appeared initially to be located in the inner tube at +63° moved progressively toward the outer wall (+30°), reaching it at 0° and crossing it at -20°, then moved back to the original location with further tilting (-55°). Figure S3 shows the 3D (X, Y and Z directions) images of the CNF/HPANI-Pt of a small part of Figure 9b (enclosed by a rectangle) from the reconstruction. An interesting aggregation of Pt particles (enclosed by a hexagon) is shown in three different projections (X, Y and Z), and all the three projections also clearly show that the aggregation of the particles is within the inner tube.

To study the accessibility of the inner tube of the CNF/HPANI-Pt catalyst support, EDS mapping of the distribution of fluorine (F) was done on the catalyst sample deposited on a copper grid in the TEM (JEOL 2200FS). From Figure S4, it can be observed that the inner tube of the catalyst support can be accessed by the Nafion electrolyte since a strong fluorine signal was detected within the inner tube. The Nafion solution was the only source of F amongst the reagents used. As the electrochemical oxidation of methanol always takes place at the interface between the Pt particles and the Nafion ionomer, these particles in the inner tube accessible to the Nafion ionomer electrolyte can still enable the catalytic oxidation of methanol. Moreover, the majority of the HPANI/CNF has an open-ended hollow structure, which means that the internal surface of the hollow tubes is readily accessible to the electrolyte and fuel. Thus, CNF/HPANI-Pt shows higher electrochemical catalytic performance to the oxidation of methanol than the CNF/PANI-Pt catalyst.

## Conclusions

A facile process for the synthesis of CNF/PANI core/shell composite was developed. Carbonization of the composite produces core/shell carbon fibers with N-doping at the surface, which has been confirmed by XRD, FTIR, Raman and EDS studies. TEM tomography shows that the Pt nanoparticles were dispersed both on the inner surface and on the outer surface of the hollow N-doped carbon nanofibers. EDS mapping confirmed that the particles on the inner surface of the tube are still accessible by the Nafion ionomer electrolyte. Hence, carbonization provides significant improvements (approximately seven times) in their catalytic performance as catalyst supports for methanol electrochemical oxidation.

## Abbreviations

CNF: carbon nanofiber; GC: glassy carbon; HPANI: carbonized polyaniline; PANI: polyaniline; SCE: saturated calomel electrode; SEM: scanning electron microscopy; TEM: transmission electron microscopy.

## Competing interests

The authors declare that they have no competing interests.

## Authors' contributions

CZ conducted the catalyst study, vibrational spectroscopy tests and morphology characterization, participated in the sequence data analysis and drafted the manuscript. ZL, XD and YY conceived and designed the research plan. ZL and XD performed the statistical analysis and corrected the draft for important intellectual content. DRGM provided technical support on the tomography study of the catalysts and provided further input to the draft. ZL and SR financially supported the spectroscopy tests and revised the draft manuscript. YWM drafted, revised and finalized the manuscript. All authors read and approved the final version of the manuscript.

## Supplementary Material

Additional file 1 **Supporting information**. This file contains Figure S1, TEM micrographs and EDS line scan of CNF/PANI and CNF/HPANI; Figure S2, EDS of CNF/HPANI; Figure S3, TEM tomography reconstructed images of CNF/HPANI-Pt catalyst of a small part of the same sample in Figure 9b; Figure S4, TEM-EDS elemental mapping of C, Pt and F of the CNF/HPANI-Pt with the Nafion ionomer electrolyte; and Table S1, EDS data of CNF/HPANI. Click here for file
