# Supplementary material for: Hollow nitrogen-containing core/shell fibrous carbon nanomaterials as support to platinum nanocatalysts and their TEM tomography study
Source: Nanoscale Res Lett. 2012 Mar 2;7(1):165. doi: 10.1186/1556-276X-7-165 (PMC3311096; doi:10.1186/1556-276X-7-165)
Supplement: Additional file 1 — Supporting information. This file contains Figure S1, TEM micrographs and EDS line scan of CNF/PANI and CNF/HPANI; Figure S2, EDS of CNF/HPANI; Figure S3, TEM tomography reconstructed images of CNF/HPANI-Pt catalyst of a small part of the same sample in Figure 9b; Figure S4, TEM-EDS elemental mapping of C, Pt and F of the CNF/HPANI-Pt with the Nafion ionomer electrolyte; and Table S1, EDS data of CNF/HPANI. [file 1556-276X-7-165-S1.DOC]

Supporting information:


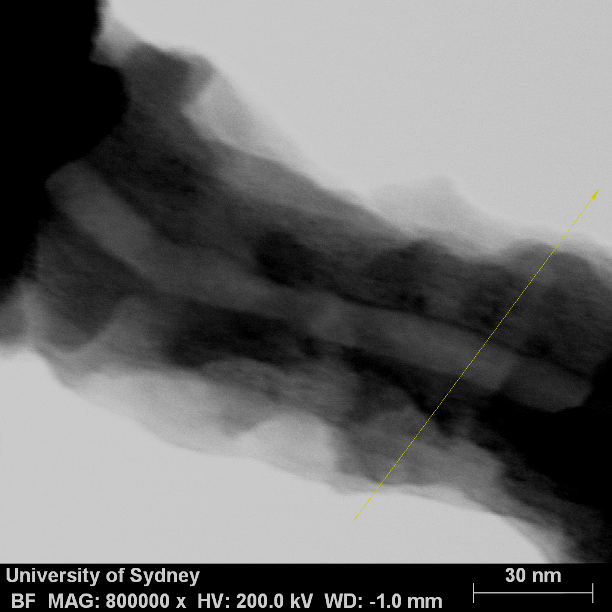

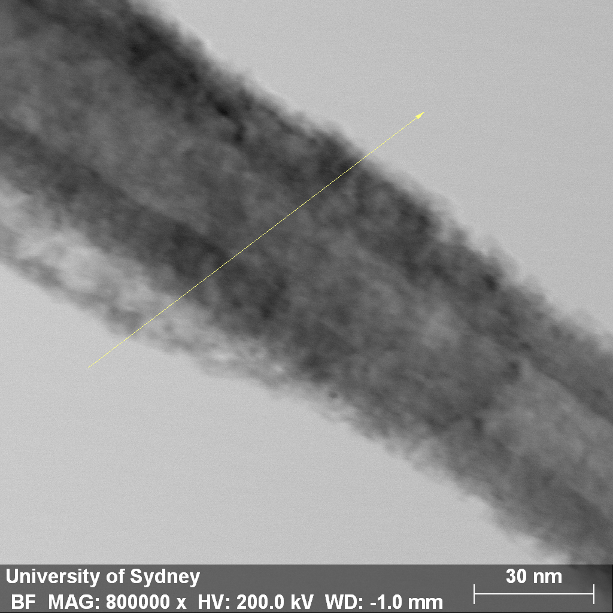


a

b


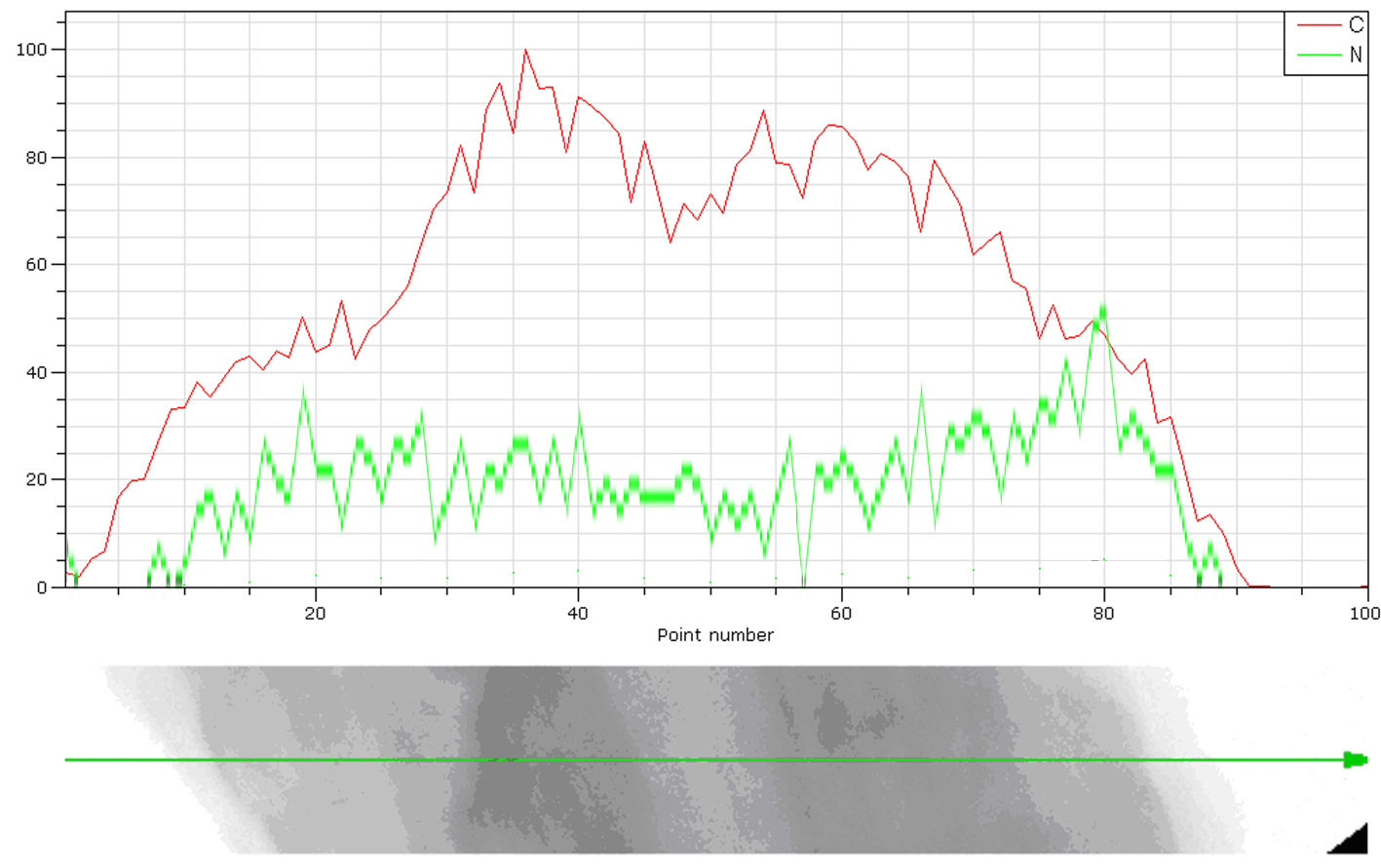

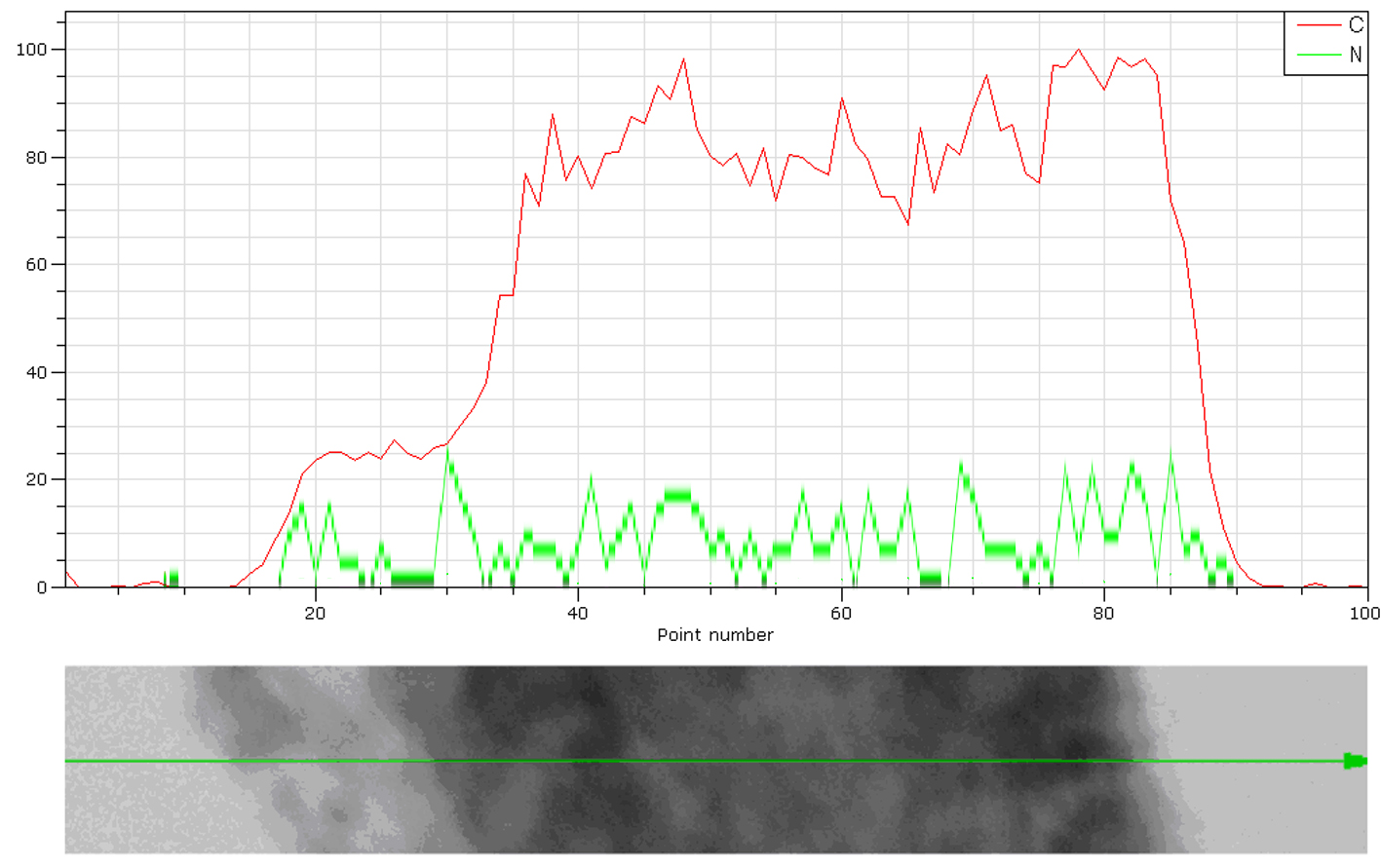


c

d

Figure S1. TEM micrographs and EDS (N x 10) line scan of (a,c) CNF/PANI and (b,d) CNF/HPANI.

Figure S2. EDS of CNF/HPANI.

**Table S1 EDS data**: CNF/HPANI (Objects 444)

Element Series norm. C Atom. C Error (1 Sigma)

[wt.%] [at.%] [wt.%]

-------------------------------------------------

Carbon K-series 99.53 99.62 3.22

Nitrogen K-series 0.24 0.20 0.05

Oxygen K-series 0.23 0.17 0.05

-------------------------------------------------

Total: 100.00 100.00


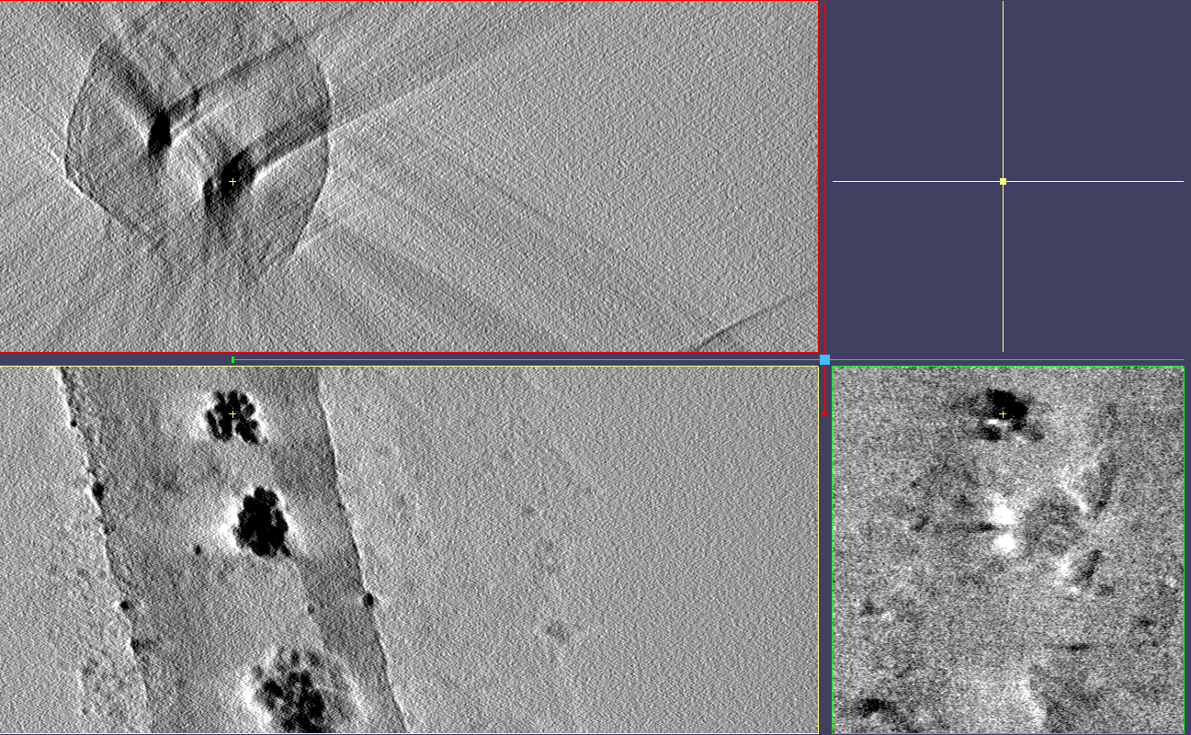


Z

Y

X

Figure S3. TEM tomography reconstructed images of CNF/HPANI-Pt catalyst of a small part of the same sample in Figure 9b in three directions (that is, the XYZ windows).


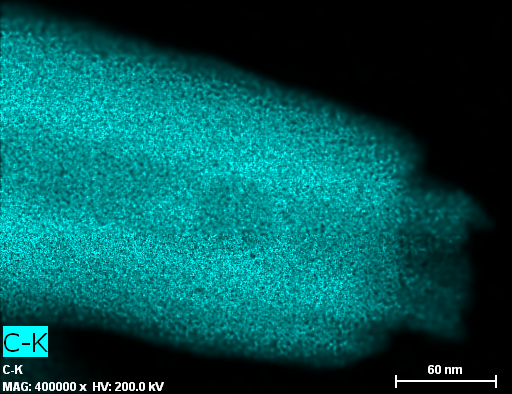


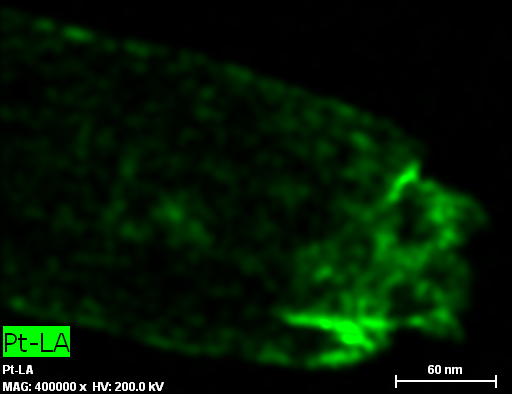


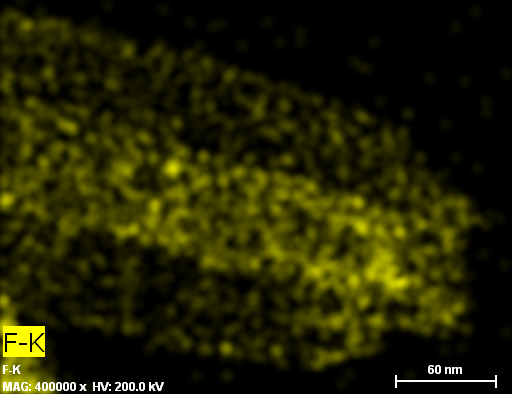


Figure S4. TEM-EDS elemental mapping of C, Pt and F of the CNF/HPANI-Pt with Nafion ionmer electrolyte.
